# COVID-19: public health management of the first two confirmed cases identified in the UK

**DOI:** 10.1017/S0950268820001922

**Published:** 2020-08-28

**Authors:** B. Holden, A. Quinney, S. Padfield, W. Morton, S. Coles, P. Manley, A. Wensley, C. Hutchinson, P. J. Lillie, C. J. A. Duncan, M. L. Schmid, A. Li, K. Foster, S. Anaraki, G. Dabrera, M. Zambon, G. J. Hughes, M. Gent

**Affiliations:** 1 Public Health England Yorkshire and the Humber, Health Protection Team, Leeds, UK; 2 Field Epidemiology, Field Service, National Infection Service, Public Health England, Leeds, UK; 3 Field Epidemiology, Field Service, National Infection Service, Public Health England, Newcastle, UK; 4 Public Health England Yorkshire and the Humber, Communications Team, Leeds, UK; 5 Hull University Teaching Hospitals NHS Trust, Department, Hull, UK; 6 Department of Infection and Tropical Medicine, The Newcastle Upon Tyne Hospitals NHS Foundation Trust, Newcastle, UK; 7 Department of Microbiology, The Newcastle Upon Tyne Hospitals NHS Foundation Trust, Newcastle, UK; 8 Public Health England North East, Health Protection Team, Newcastle, UK; 9 Public Health England North East and North Central London, Health Protection Team, London, UK; 10 Public Health England National Infection Service, Colindale, UK

**Keywords:** Coronavirus, COVID-19, emerging infections, respiratory infections

## Abstract

We report key learning from the public health management of the first two confirmed cases of COVID-19 identified in the UK. The first case imported, and the second associated with probable person-to-person transmission within the UK. Contact tracing was complex and fast-moving. Potential exposures for both cases were reviewed, and 52 contacts were identified. No further confirmed COVID-19 cases have been linked epidemiologically to these two cases. As steps are made to enhance contact tracing across the UK, the lessons learned from earlier contact tracing during the country's containment phase are particularly important and timely.

## Introduction

In December 2019, an outbreak of a novel coronavirus disease (COVID-19) caused by the SARS-CoV-2 virus was reported in Wuhan, China [1]. The severity of this respiratory disease varies from a mild self-limiting condition, to a serious and potentially fatal illness [2]. The first two confirmed cases of COVID-19 were detected in the UK on 30 January 2020 [3]. This article describes the public health management of those cases. The learning discussed may be relevant to the management of future cases of any high consequence infectious disease (HCID) as well as COVID-19 cases and clusters.

As of 19 July 2020, there had been a cumulative total of 254 519 laboratory-confirmed cases of COVID-19 diagnosed in England. Weekly numbers of new laboratory-confirmed cases (by specimen date) peaked at 27 907 in the week commencing on 23/04/2020 and has declined since then to 3693 in the week commencing 15/07/20 [4]. The highest cumulative rate since 30 January 2020 occurred in the North West of England (588/100 000 population) and the lowest in the South West of England (228/100 000). As of 7 July 2020, where ethnicity was known, the largest numbers of cases occurred in the White ethnic group (81.5%), followed by the Asian/Asian British (10.0%) and Black/Black African/Black Caribbean/Black British group (4.6%). The highest number of cumulative deaths occurred in the 80 years and older age group (23 364).

The UK pandemic response started with a contain phase and then transitioned to a delay phase [5]. During the contain phase, over 5000 people were followed-up as contacts in England alone [6]. During the delay phase, the UK implemented social and physical distancing measures, similar to many countries [7]. In addition, persons defined as extremely vulnerable to serious illness were asked to stay at home and minimise interactions (‘shielding’) [8]. The NHS test and trace service facilitates testing of persons with symptoms compatible with COVID-19 and undertakes contact tracing of those who test positive [9].

The index case (Case 1) arrived in the UK in late January from the Hubei province in China. They travelled with another person (co-traveller) and both were asymptomatic during the journey. Three days after arrival in the UK; Case 1 developed a fever, dry cough and sore throat. Case 2, a UK resident and close contact of Case 1, developed a fever and malaise 2 days later.

Case 1 and Case 2 made contact with the National Health Service. Discussion with local health protection services identified both cases to be at risk of COVID-19. Both cases were admitted to an infectious diseases unit for isolation, assessment and diagnostic sampling.

Testing and sequence confirmation using methods described by Corman *et al*. was carried out the same day, and SARS-CoV-2 infection was confirmed [10]. Rapid laboratory support to confirm cases of COIVD-19 was essential in a complex tracing arrangement.

## Further investigation and contact risk assessment

**Fig. 1.**
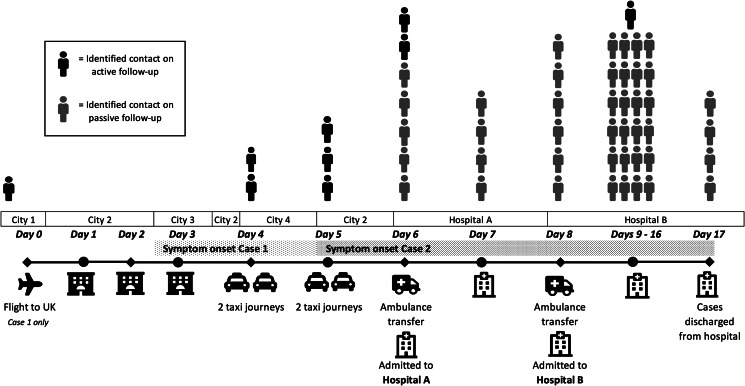
Timeline detailing key settings and exposures where contact tracing took place; and the number of identified contacts of the first two confirmed cases of COVID-19 identified in the UK, January 2020. Please note: *Case 2* is not included as an identified contact of *Case 1* in this timeline.

Case 1 and Case 2 were interviewed with a translator to identify all contacts between symptom onset and hospital admission. Relevant settings and identified contacts are summarised in Figure 1. Case 1 and Case 2 had limited social contact with others whilst symptomatic. Please note that Case 2 is not included as a contact in Figure 1 as the timeline groups all contacts of Case 1 and Case 2.

Case 1 and Case 2 were admitted to hospital on 29 January 2020. This was after following the recommended guidance for initial telephone contact with healthcare services and a planned admission as possible cases.

Potential exposures for both cases were reviewed, and 52 contacts were identified (excluding Case 2 but including the co-traveller of Case 1). This included 45 healthcare workers (HCWs). As the cases were managed as possible cases, the majority (43) of these HCWs had low-risk exposures, and required only passive follow-up, while two required active follow-up on a precautionary basis. Those receiving active follow-up were contacted daily for 14 days to ensure they remained well. Those under passive follow-up were provided with information and requested to contact PHE if they became symptomatic. A further six contacts in the community were identified with different categories of exposure, as described in Table 1.

**Table 1.** Categorisation and number of identified contacts of Case 1 and Case 2 (numbers do not include Case 2 as an identified contact of Case 1)

Category	Description	Follow-up	Self-isolation	Number of identified contacts
A	Household contact – living or spending significant time in the same household	Active follow-up for 14 days after last exposure	Yes	1
B	Persons in healthcare settings (e.g. healthcare workers, visitors) who **have not** worn recommended PPE or laboratory workers who **have not** used appropriate laboratory precautions	Active follow-up for 14 days after last unprotected exposure	Yes	2
C	Persons in healthcare settings who have worn recommended PPE	Passive follow-up for 14 days after last exposure	No	43
D	**For any other (non-aircraft) exposure not satisfying categories A-C:** Direct contact or face-to-face contact with case, e.g. talking, being coughed on **for** any length of time	For exposures in this category **without recommended PPE:** Active follow-up for 14 days after last unprotected exposure. For exposures in this category **with recommended PPE**: Passive follow-up for 14 days after last exposure	Active follow-up group: Yes Passive follow-up group: No	6
**Total contacts:**				**52**

Of all contacts under follow-up (excluding Case 2), two developed mild symptoms compatible with COVID-19. No identified contacts (excluding Case 2) tested positive for SARS-CoV-2.

From the start of the investigation up until hospital discharge, the co-traveller of Case 1 remained asymptomatic for COVID-19. As a close contact, they were regularly tested by PCR on upper respiratory specimens for SARS-CoV-2. All tests were negative, despite them being a close household contact of both cases. Serological testing was not undertaken at the time of follow-up.

Accommodation sites where the cases had residential-type exposures were assessed as being potentially higher risk. If not previously cleaned, these were restricted from use for a minimum of 5 days after last use. This was in order to minimise the risk to others and based on the limited available evidence about environmental contamination at the time [5–7, 11–13].

## Virology

Whole-genome sequencing data were made available for Case 1 and Case 2 and uploaded to GISAID [14]. Although no further confirmed COVID-19 cases have been linked epidemiologically to these two cases, this assertion may yet be tested genomically. Supported by the UK government, the COVID-19 Genomics UK (COG-UK) consortium has been created to deliver large-scale and rapid whole-genome virus sequencing to guide UK public health interventions and policies [15].

Biological materials from the first cases have given rise to an infectious virus isolate which has been shared safely with international partners. This virus isolate is being used as a prototype virus strain internationally and will be important in the development of diagnostics and potential vaccines.

## Discussion

We report the public health management of the first two confirmed cases of COVID-19 in the UK. The first case imported, and the second associated with probable person-to-person transmission within the UK. This response was part of the containment phase of the UK government COVID-19 response.

This early experience reinforces key learning from elsewhere in the pandemic, such as the high risk of transmission to close contacts as well as the lower risk to HCWs when infection prevention control measures are appropriately followed.

A small number of contacts had exposures with potentially higher risks such as those cleaning environments which were potentially contaminated. These groups were placed under active follow-up but did not develop COVID-19. Although the overall numbers were small, this contributes further to the evidence base needed to inform future public health strategies. Initial advice was based on the precautionary principle, to prevent members of the public from any potential risk of exposure to the SARS-CoV-2 virus.

Managing environments which cases had inhabited in a residential-type setting was a challenge. In the absence of significant published data on environmental contamination related to cases at that stage of the pandemic, this required application of the precautionary principle based on previous experience from high consequence coronaviruses, to inform a practical approach. These cases brought to the surface the importance of data on environmental survival of viruses to inform practical decontamination guidance for future public health management. This demonstrates the importance of detailed risk assessments for the first identified cases in any novel disease outbreak.

Current technology provides further information about possible exposure settings, and opens the door to previously unused methods of identifying potential contacts [16]. In this instance, taxi drivers were identified through journey records held within the case's online taxi booking application. A data-sharing agreement was agreed with the taxi company and Public Health England (PHE) were able to contact the taxi drivers to recommend self-isolation and commence active follow-up. However, these additional information sources can only supplement other data sources such as case questionnaires rather than act as a direct replacement.

This experience also reinforced previous learning from other diseases, in relation to the reliance on self-reporting and individual's recollection of face-to-face interactions [17]. To maximise contact identification for these cases, repeat interviews were conducted to confirm information gathered and contextual prompts were used.

There was significant national, regional and local media and social media interest regarding the public health management of the first two confirmed cases identified in the UK. PHE communications colleagues provided a 24/7 media handling service throughout the response in liaison with the relevant local government authority. Due to the high-profile nature of the first confirmed cases of COVID-19 in the UK, the initial media response was led nationally, by the Department of Health and Social Care.

Contact tracing was complex and fast-moving. Additional challenges were encountered through media and national interest and having to rapidly review the available evidence and develop scientific consensus to inform actions. Despite these challenges, no further confirmed cases linked to Case 1 and Case 2 were identified in the UK.

## Conclusions

The manuscript discusses the public health management of the first two confirmed cases of COVID-19 identified in the UK. We believe that the learning discussed may be relevant to the management of future cases of any HCID as well as COVID-19 cases and clusters. As steps are made to renew contact tracing across the UK, the lessons learned from contact tracing during the country's containment phase are especially important.

Our manuscript examines the complex and fast-moving contact tracing efforts, in addition to the challenges encountered at an early stage of the epidemic. We have also described the epidemiological links between the identified cases, and refer to the future work that may be pursued to test epidemiological findings using genomics. To date, there has not been any published data regarding contact tracing and public health management of COVID-19 in England. This would be the first publication of such data.

## Data Availability

The data analysed during this study are not publicly available due to a need to protect individual's anonymity. These data are confidential, but fully anonymised data may be available from the corresponding author on reasonable request.
